# The optimal radiofrequency temperature in radiofrequency thermocoagulation for idiopathic trigeminal neuralgia: A cohort study

**DOI:** 10.1097/MD.0000000000004103

**Published:** 2016-07-18

**Authors:** Yuan-Zhang Tang, Li-Qiang Yang, Jian-Ning Yue, Xiao-Ping Wang, Liang-Liang HE, Jia-Xiang NI

**Affiliations:** Department of Pain Management, Xuanwu Hospital of Capital Medical University, Beijing, China.

**Keywords:** pain, radiofrequency temperature, radiofrequency thermocoagulation, trigeminal nerve, trigeminal neuralgia

## Abstract

**Objective:**

Our previous study evaluated the effectiveness and safety of radiofrequency thermocoagulation (RFT) of trigeminal gasserian ganglion for idiopathic trigeminal neuralgia (ITN). The aim of this study was to evaluate the optimal radiofrequency temperature of computed tomography (CT)-guided RFT for treatment of ITN.

**Methods:**

A retrospective study of patients with ITN treated with a single CT-guided RFT procedure between January 2002 and December 2013. Patients were divided into ≤75 °C, 75 °C, and ≥80 °C groups according to the highest radiofrequency temperature used. Pain relief was graded from poor to excellent, and facial numbness/dysesthesia from I (absent) to IV (most severe).

**Results:**

A total of 1161 RFT procedures were undertaken in the 1137 patients. The mean follow-up time was 46 ± 31 months. There were no significant differences in the rate of excellent pain relief according to the radiofrequency temperature used. However, more patients experienced with no facial numbness or facial numbness gradually resolved and those patients treated at 75 °C had a lower rate of grade IV facial numbness/dysesthesia than other groups.

**Conclusions:**

The optimal radiofrequency temperature to maximize pain relief and minimize facial numbness or dysesthesia may be 75 °C, but this requires confirmation.

## 1 Introduction

Idiopathic trigeminal neuralgia (ITN) is an uncommon pain disease, mean annual incidence of 12.6 per 100,000 person years.^[1]^ It is characterized by excruciating pain, which seriously impairs the quality of life of those with the condition. It should be distinguished from symptomatic trigeminal neuralgia (STN), by the absence of any structural lesion affecting the trigeminal nerve. The pathogeny of ITN is complicated and undefined. Although most favor the vascular compression hypothesis.^[2]^ However, Taha and Tew^[3]^ reported that around 15% of patients with ITN lacked evidence of significant vascular compression; Devor et al^[4]^ considered that compression and demyelination of sensory nerves generally causes numbness or loss of vibration sense, rather than pain.

Fortunately, whatever the cause, there are effective treatments for ITN, and a number of treatment guidelines have been published.^[5–7]^ Treatment options include drugs and interventional procedures. Anticonvulsant drugs are generally used first line. There are several neurosurgical procedures available to treat patients if drug treatment is not tolerated or is ineffective. These include ablation technique and microvascular decompression (MVD).

Among the ablation techniques, radiofrequency thermocoagulation (RFT) of trigeminal gasserian ganglion is reported to give higher success rates than other ablation techniques, such as glycerol rhizotomy or stereotactic radio suigery.^[3,8]^ We also found that RFT is a practical treatment option in treating recurrent TN after MVD.^[9]^ Although the RFT has now widespread application in the treatment of TN, different temperatures (55–90 °C) in operation determined by doctor's experience,^[10–12]^ the optimal temperature to maximize pain relief and minimize complications has no unified standard.

RFT of the trigeminal ganglion to treat ITN was first reported in 1974^[13]^ and was introduced to China (by Professor Ni) in 2000. Since 2002, we have performed computed tomography (CT)-guided RFT and have reported promising preliminary results.^[9,14–17]^ All RFT procedures in our unit are now CT guided. Here we report a retrospective study analyzing the optimal radiofrequency temperature in patients with ITN treated with a single RFT procedure.

## 2 Materials and methods

### 2.1 Patients

The study was approved by the institutional review board and local ethics committee. Written informed consent was obtained from each patient before the RFT procedure. Inclusion and exclusion criteria for patients and the methods of follow-up were according to our previous report.^[16]^

### 2.2 RFT procedures

We have previously described our RFT procedure in detail.^[9,15,16]^

### 2.3 Data collection

Demographic information, the duration of symptoms preprocedure, the duration of follow-up postprocedure, the trigeminal nerves and divisions treated, the temperatures used during RFT, and the census point for data collection were obtained from the medical records and follow-up data.

We initially divided the patients into 5 groups, according to the highest temperature used during the RFT procedure: 65 °C, 70 °C, 75 °C, 80 °C, and 85 °C. However, because there were only 8 and 5 patients treated with temperatures of 65 °C and 85 °C_,_ respectively; we later allocated the patients into 3 groups for data analysis: ≤75 °C, 75 °C, and ≥80 °C.

Pain intensity evaluation, pain relief (“excellent,” “good,” “fair,” and “poor”), and facial numbness/dysesthesia outcomes classification (grade I–IV) is studied using the methods mentioned in the previous paper.^[14,16]^

### 2.4 Statistical analysis

Statistical analysis was performed using the statistical package for the social sciences version 17.0 (SPSS, Chicago, IL). Kaplan–Meier curves were used to determine the percentage of patients that were free of pain and free of facial dysesthesia after RFT. The log-rank test was used to compare survival curves. The censor point for data collection was either: the last contact with the patient; the time of any subsequent surgery; or death. For statistical purposes, in patients who died, the duration of pain relief was assessed as the time from the procedure to the time of death. For patients who were lost to follow-up, data obtained at the time of the last contact was used. If a second RFT procedure on the same nerve during the same hospital stay was required, this was considered as a single procedure for statistical purposes. If bilateral RFT procedures were undertaken on the same patient, this was treated as 2 procedures for statistical purposes. A *P* value of < 0.05 was considered statistically significant.

## 3 Results

A total of 1161 RFT procedures were undertaken in the 1137 patients. The following information is shown in Table 1. A total of 121patients (11%) were lost to follow-up, which was not predicted by either the degree of postprocedure pain relief or the complication rate. However, 89 patients (7.7%) died from causes unrelated to the RFT procedure, and 84 patients (7.2%) had a second RFT procedure on the same trigeminal nerve during the same hospital stay. The need for this appeared to be related to a lack of a clear response to stimulation during the initial procedure. According to the original radiofrequency temperature: 247 (21.3%) patients, including 8 (0.7%) patients treated with 65 °C radiofrequency and 239 (20.6%) patients with 70 °C, were enrolled in the ≤70 °C group; 790 (68%) patients treated with 75 °C were enrolled in the 75 °C group; 124 (10.7% patients), including 119 (10.3%) patients treated with 80 °C and 5 (0.4%) patients with 85 °C, were enrolled in the ≥80 °C group.

**Table 1:**
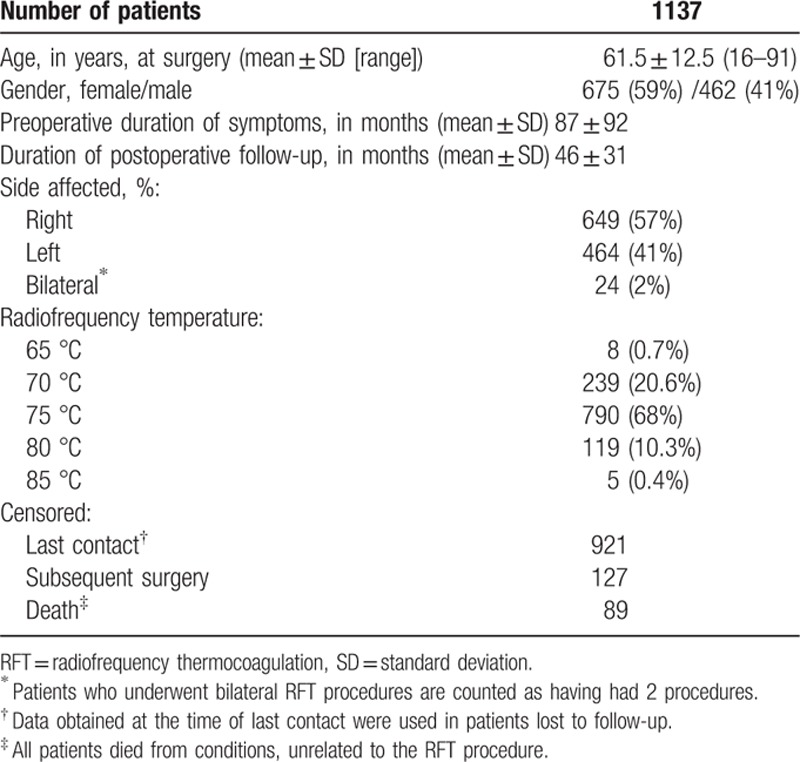
Demographic, clinical, and RFT data on patients studied.

### 3.1 Pain relief according to different radiofrequency temperatures

**Figure 1:**
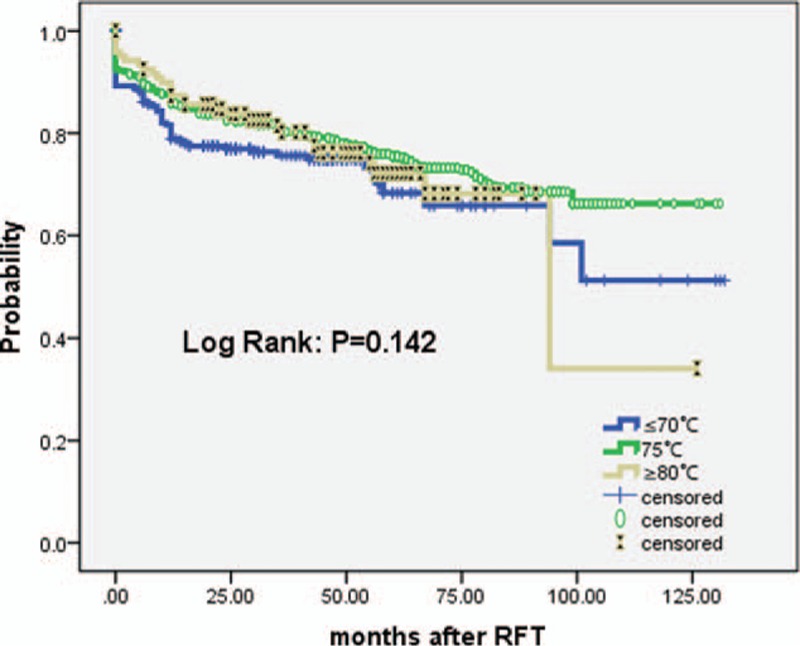
Actuarial Kaplan–Meier curve comparing the long-term outcomes of patients pain-free off medications according to different radiofrequency temperatures (≤70 °C, 75 °C, and ≥80 °C) in the treatment of patients with ITN. ITN = idiopathic trigeminal neuralgia.

The mean ± SD NRS scores before RFT in patients treated using temperatures of ≤70 °C, 75 °C, and ≥80 °C were 7.75 ± 1.44, 7.78 ± 1.37, and7.83 ± 1.37, respectively (*P* > 0.05). At follow-up of the census point, the respective NRS scores were: 0.88 ± 2.21, 0.61 ± 1.88, and 0.57 ± 1.47 (*P* > 0.05), which indicated the mean pain intensity of all patients has been alleviated significantly. Figure 1 shows the Kaplan–Meier actuarial curve for patients who achieved initial excellent pain relief, according to the temperature used during RFT, the log-rank test (for each stratum) did not show any statistical difference between groups (*P* > 0.05), which indicated that excellent pain relief could be obtained as long as the radiofrequency temperature used >65 °C.

### 3.2 Facial numbness/dysesthesia according to different radiofrequency temperatures

Most patients reported facial numbness immediately after RFT. However, 57 patients, including 17 (30%) patients in the ≤70 °C group, 26 (45.6%) patients in the 75°C group, and 14 (24.4%) patients in the ≥89 °C group, never experienced facial numbness which was graded I. A total of 243 patients reported that initial facial numbness gradually resolved and had gone totally after mean ± SD duration of 31.4 ± 14.9 weeks which showed in Table 2. More patients in the 75 °C group experienced with no facial numbness (grade I 45.6%) and facial numbness gradually resolved (grade II 60% and grade III 58.9%) than other 2 groups.

**Table 2:**
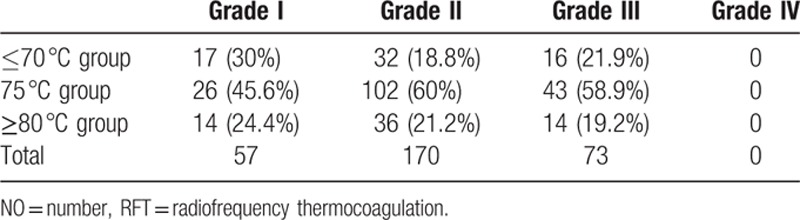
Patients experienced no facial numbness or facial numbness gradually resolved after RFT in different groups (NO. %).

**Figure 2:**
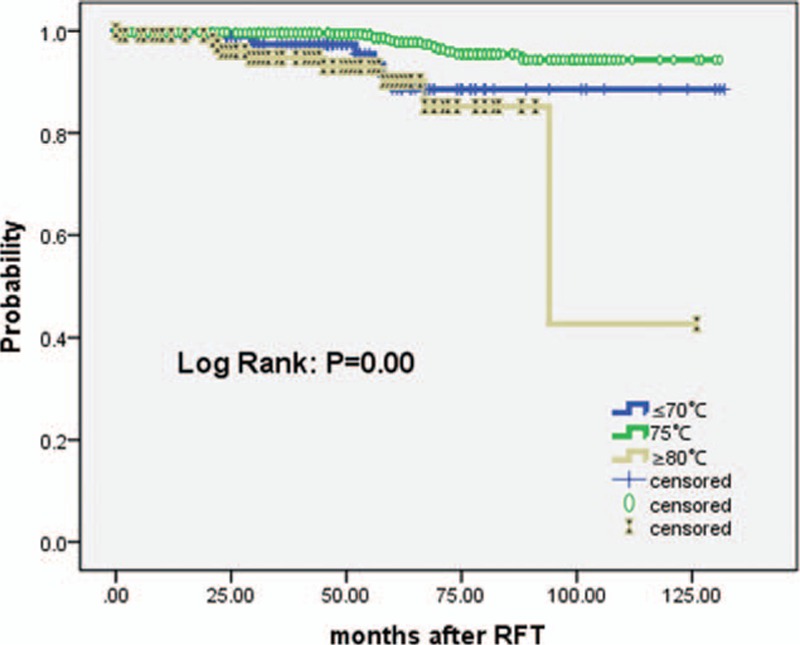
Actuarial Kaplan–Meier curve comparing the long-term outcomes of patients painful dysesthesia free according to the different radiofrequency temperatures (≤70 °C, 75 °C, and ≥80 °C) in the treatment of patients with ITN. ITN = idiopathic trigeminal neuralgia.

The degree of facial numbness in those who continued to experience it varied. It was graded II, III, and IV in 465 (54%), 343 (39.8%), and 53 (6.2%) patients, respectively. Grade IV facial numbness was reported by 19 of 247 patients (7.7%), 16 of 790 patients (2%), and 18 of 124 patients (14.5%) who had been treated with RFT using temperatures of ≤70 °C, 75 °C, and ≥80 °C, respectively. We further analyzed using a Kaplan–Meier actuarial curve and setting the event as grade IV facial numbness (Fig. 2). Those treated with RFT using a temperature of 75 °C had a lower incidence of dysesthesia (grade IV facial numbness) when compared to those treated using temperatures of ≤70 °C or ≥80 °C (*P* < 0.01). All patients who experienced facial numbness reported it to lessen with time.

### 3.3 Adverse effects

Other complications including Masseter muscle weakness occurred in 91 patients (8%), Corneitis in 29 patients (2.6%), diplopia in 14 patients (1%); low pressure headache in 2 patients (0.2%), experienced ptosis in 8 patients (0.7%); difficulty with mouth opening in 5 patients (0.4%); and hearing loss in 5 patients (0.4%). These complications maybe related to the operation technique, other than radiofrequency temperature. All these problems tended to improve with time. No other cranial nerve problems occurred, there were no cases of carotid-cavernous fistula, and there was no procedure-related mortality.

## 4 Discussion

The ideal surgical treatment for trigeminal neuralgia should have a high success rate in terms of control of pain, be minimally invasive, have few complications, and be well accepted by patients. No currently available technique meets all of these requirements; each has advantages and disadvantages. RFT satisfies the first 3 of these aims. A relatively high incidence of adverse effects is its principle drawback, limiting its more widespread use.

RFT of the trigeminal gasserian ganglion has been popularized since 1974, as an effective treatment which is less invasive than some alternatives.^[13]^ The heat produced by the RPF needle is thought to selectively destroy the Aδ and C pain fibers by thermocoagulation at temperatures above 65 °C.^[18]^ This can prevent both triggering of pain and the intensity of pain, but can also cause numbness and other problems.^[8,19]^

Postoperative facial numbness is common following RFT. It usually accompanies the relief from pain. Temperatures of >65 °C are known to destroy nerve fibers, with the Aδ and C nociceptive fibers, being destroyed at lower temperatures than are required to destroy the Aα and Aβ tactile fibers. However, to date, selection of radiofrequency temperatures to selectively destroy nociceptive and preserve tactile fibers has not been reported. Generally temperatures of between 55 °C and 85 °C are used during RFT to treat trigeminal neuralgia.^[12,20]^ Our previous study reported that complete pain relief was less common in those in whom no facial numbness, or in whom it was transient, indicated that a degree of facial numbness may be inevitable if long-term relief of pain symptoms is to be achieved. It may be more logical to view facial numbness as a predictable side effect of RFT, rather than as an unexpected complication.

We have analyzed the long-term effective rate of different branches of these 1137 ITN patients after single radiofrequency thermocoagulation in our previous report.^[16]^ In this study, we further explored the outcome, both in terms of pain relief and facial numbness/dysesthesia, of RFT according to the temperature used during the procedure. We found no significant difference in the rate of pain relief according to the temperature used, although more patients experienced with no facial numbness or facial numbness gradually resolved in 75 °C temperatures of 70 °C and below, or of 80 °C and above were more likely to suffer severe facial numbness with dysesthesia than those treated at 75 °C. Facial numbness, particularly dysesthesia, is a serious side effect of RFT that affects the quality of patients’ lives. Given our findings, we recommend that when using RFT to treat ITN, the temperature used should generally be 75 °C, as this seems to optimize pain relief while minimizing the risk of facial numbness and painful dysesthesia.

We do not have an explanation why the outcome of RFT is better when using a temperature of 75 °C, rather than with higher or lower temperatures. However, the higher the temperature used, the greater the size of the coagulum.^[21,22]^ We suspect that the choice of which temperature to use during RFT is influenced by the voltage required during motor and sensory stimulations before the thermocoagulation procedure. If >0.1 V produces paresthesia and/or twitching, the operator is likely to use a temperature of ≤70 °C. If 0.1 to 0.3 V is required, 75 °C is likely to be chosen, whereas if >0.3 V is required, a temperature of ≥80 °C is likely to be used. The voltage required to produce effective stimulation reflects the distance of the needle tip from its target nerve tract in the gasserian ganglion; the smaller the distance the less voltage required. This needs to be considered when choosing a voltage which is likely to be effective, while minimizing the risk of painful dysesthesia. We hope our findings will provoke further research into the optimal temperature to use according to different stimulation voltages during RFT.

## 5 Conclusions

From our data, the optimal RFT temperature to achieve long-lasting pain relief while minimizing facial numbness and painful dysesthesia may be 75 °C, but this requires further study.
